# Molecular and Structural Aspects of Clinically Relevant Mutations of SARS-CoV-2 RNA-Dependent RNA Polymerase in Remdesivir-Treated Patients

**DOI:** 10.3390/ph16081143

**Published:** 2023-08-12

**Authors:** Carmen Gratteri, Francesca Alessandra Ambrosio, Antonio Lupia, Federica Moraca, Bruno Catalanotti, Giosuè Costa, Maria Bellocchi, Luca Carioti, Romina Salpini, Francesca Ceccherini-Silberstein, Simone La Frazia, Vincenzo Malagnino, Loredana Sarmati, Valentina Svicher, Sharon Bryant, Anna Artese, Stefano Alcaro

**Affiliations:** 1 Dipartimento di Scienze della Salute, Campus “S. Venuta”, Università degli Studi “Magna Græcia” di Catanzaro, Viale Europa, 88100 Catanzaro, Italy; carmen.gratteri@studenti.unicz.it (C.G.); gcosta@unicz.it (G.C.); alcaro@unicz.it (S.A.); 2 Dipartimento di Medicina Sperimentale e Clinica, Campus “S. Venuta”, Università degli Studi “Magna Græcia” di Catanzaro, Viale Europa, 88100 Catanzaro, Italy; ambrosio@unicz.it; 3 Dipartimento di Scienze della vita e dell’ambiente, Università degli Studi di Cagliari, Cittadella Universitaria di Monserrato, 09124 Cagliari, Italy; antonio.lupia@unica.it; 4 Net4Science S.r.l., Università degli Studi “Magna Græcia” di Catanzaro, 88100 Catanzaro, Italy; 5 Dipartimento di Farmacia, Università degli Studi di Napoli “Federico II”, Via D. Montesano 49, 80131 Napoli, Italy; bruno.catalanotti@unina.it; 6 Dipartimento di Medicina Sperimentale, Università Tor Vergata di Roma, Via Montpellier, 1, 00133 Roma, Italy; maria.bellocchi@gmail.com (M.B.); luca.carioti@yahoo.com (L.C.); rsalpini@gmail.com (R.S.); ceccherini@med.uniroma2.it (F.C.-S.); 7 Dipartimento di Biologia, Università Tor Vergata di Roma, Via della Ricerca Scientifica, 1, 00133 Roma, Italy; simone.la.frazia@uniroma2.it (S.L.F.); valentina.svicher@uniroma2.it (V.S.); 8 Dipartimento di Medicina dei Sistemi, Università Tor Vergata di Roma, Via Montpellier, 1, 00133 Roma, Italy; vincenzo.malagnino@uniroma2.it (V.M.); loredana.sarmati@uniroma2.it (L.S.); 9 Inte:Ligand GmbH, Mariahilferstrasse 74B/11, 1070 Vienna, Austria; bryant@inteligand.com

**Keywords:** SARS-CoV-2, RNA-dependent RNA polymerase, mutations, remdesivir, molecular dynamics, PCA, pharmacophore

## Abstract

(1) Background: SARS-CoV-2 RNA-dependent RNA polymerase (RdRp) is a promising therapeutic target to fight COVID-19, and many RdRp inhibitors nucleotide/nucleoside analogs, such as remdesivir, have been identified or are in clinical studies. However, the appearance of resistant mutations could reduce their efficacy. In the present work, we structurally evaluated the impact of RdRp mutations found at baseline in 39 patients treated with remdesivir and associated with a different degree of antiviral response in vivo. (2) Methods: A refined bioinformatics approach was applied to assign SARS-CoV-2 clade and lineage, and to define RdRp mutational profiles. In line with such a method, the same mutations were built and analyzed by combining docking and thermodynamics evaluations with both molecular dynamics and representative pharmacophore models. (3) Results: Clinical studies revealed that patients bearing the most prevalent triple mutant P323L+671S+M899I, which was present in 41% of patients, or the more complex mutational profile P323L+G671S+L838I+D738Y+K91E, which was found with a prevalence of 2.6%, showed a delayed reduced response to remdesivir, as confirmed by the increase in SARS-CoV-2 viral load and by a reduced theoretical binding affinity versus RdRp (ΔGbind_WT_ = −122.70 kcal/mol; ΔGbind_P323L+671S+M899I_ = −84.78 kcal/mol; ΔGbind_P323L+G671S+L838I+D738Y+K91E_ = −96.74 kcal/mol). Combined computational approaches helped to rationalize such clinical observations, offering a mechanistic understanding of the allosteric effects of mutants on the global motions of the viral RNA synthesis machine and in the changes of the interactions patterns of remdesivir during its binding.

## 1. Introduction

Coronavirus disease 2019 (COVID-19), caused by the virus SARS-CoV-2, has emerged as one of the most widespread and devastating pandemics in recorded history [1]. To mitigate the impact of the pandemic, intense efforts are being put in place by researchers from academia and the pharmaceutical industry in order to develop diagnostic, therapeutic, and vaccine candidates. Moreover, the scientific community has been focusing on understanding the complex biology underlying the viral infection through structure-function studies of different SARS-CoV2 proteins [2]. Among them, the RNA-dependent RNA polymerase (RdRp) is crucial for the replication and transcription of SARS-CoV-2 RNA genome, and it represents an important target for the development of antiviral drugs against coronaviruses [3].

**Figure 1:**
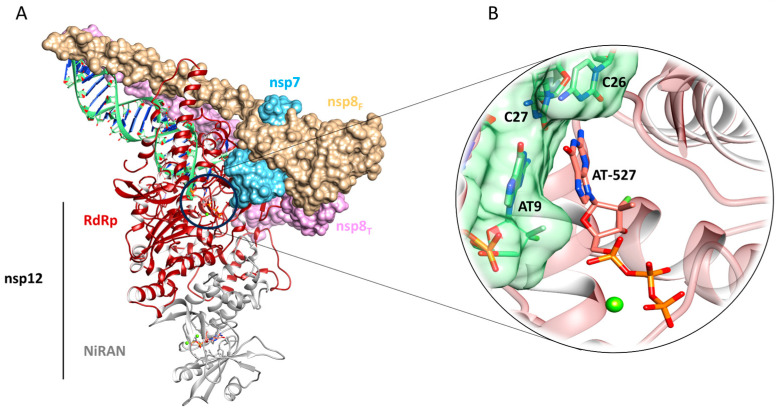
(**A**) General overview of the SARS-CoV-2 polymerase model, with the nonstructural protein Nsp7, Nsp8, and Nsp12, and the RNA (PDB ID: 7ED5). (**B**) Focus on the RdRp active site with the original complexed ligand AT-527.

Structures of RdRp have been reported for SARS-CoV-1 [4] and SARS-CoV-2 [5], and they provide insights into the mechanisms of RNA-dependent RNA synthesis [6]. The availability of structural details also allows rationalization of the molecular processes underlying the antiviral activity of the compounds targeting RdRp. Specifically, RdRp, also defined as Nsp12 (Nonstructural protein 12), consists of 932 amino acids and, along with Nsp7 and Nsp8, forms a replicase complex for replication and transcription of the viral RNA genome (Figure 1). SARS-CoV-2 Nsp12 is very similar to SARS-CoV, having a 96% sequences identity, and it contains three different domains: (i) an N-terminal extension nidovirus-unique RdRp-associated nucleotidyltransferase (NiRAN) domain (residues 60–249); (ii) an RdRp domain (residues 366–920); and (iii) an interface domain that connects the first ones. In the Protein Data Bank (PDB) [7], several single-particle cryogenic electron microscopy (cryo-EM) structures of SARS-CoV and SARS-CoV-2 Nsp12 have been deposited [8,9,10,11,12], including a complex of SARS-CoV-2 Nsp12 bound to Nsp7 and Nsp8 (PDB IDs: 7BTF, 6M71) and to primer-template RNAs along with remdesivir (PDB IDs: 7BV2, 6YYT) or its nucleoside analogue (PDB ID: 7ED5) (Figure 1).

The polymerase domain has a typical right-hand fold and consists of three subdomains: fingers (residues 366–581; 621–679), palm (residues 582–620; 680–815), and thumb (residues 816–920) [8]. The catalytic site, whose activity is ensured by two Mg^2+^ ions coordinated to D618, D760, and D761 residues, is in the palm subdomain [12]. Specifically, both Mg^2+^ ions allow the stabilization of the nucleoside triphosphate (NTP) phosphates, and the ribose moiety of the NTP incorporated in the nucleic acid is correctly placed within the catalytic site by means of the conserved serine at position 682 [13].

Recently, by using cryo-EM, the Nsp7-Nsp8 heterodimer was observed bound to the polymerase thumb subdomain of Nsp12 in front of the NTP entry channel (PDB ID: 7BV2, 6YYT), while the polymerase finger loop was inserted between Nsp7-Nsp8 and the thumb subdomain. The second subunit of Nsp8 is involved in crucial contacts with the Nsp12 interface domain close to the fingers’ subdomain and the RNA template-binding channel. The polymerase domain is highly stabilized by the binding of the Nsp7-Nsp8 heterodimer to the finger loop, with a consequent increase in its affinity towards template RNA. The polymerase activity is also affected by the second subunit of Nsp8, since its binding to the template RNA is responsible for an extended interaction surface, thereby holding the RNA strand in position. The binding affinity of Nsp12 to template-primer RNA was found to be significantly higher in the presence of both Nsp7 and Nsp8, thus resulting in an enhanced polymerase activity [9].

Many RdRp inhibitors are nucleotide/nucleoside analogs (NAs). They can act as antiviral compounds by causing either chain termination or through the increased/decreased rate of their incorporation in nascent RNA, leading to lethal mutations and nonviable genomes [14,15]. However, the appearance of NA resistant mutations, as well as the possible removal of the incorporated drug from RNA at 3′-end by the exonuclease (ExoN), could impair the efficacy of NAs [11,16]. In more detail, the selection of multiple mutational patterns conferring varying degrees of SARS-CoV-2 resistance to remdesivir have already been demonstrated to occur in vitro during serial passages in infected cells in the presence of the drug. Among them, multiple combinations of Nsp12-RdRp amino acid substitutions, involving V166A, S759A, V792I, C799F/R, and E802D, have been associated with a reduced sensitivity to remdesivir [17,18,19]. Above all, the RdRp mutational couple, S759A (residing in the catalytic site of the viral enzyme) and V792I, have been demonstrated to cause a >30-fold increase in the EC_50_ of remdesivir in in vitro experiments not only with SARS-COV-2 but also with the betacoronavirus, murine hepatitis virus (MHV). The overall data highlight the potential role of several RdRp mutations in modulating the response to the drug [19], and they raise the need to better explore the potential genetic pathways promoting SARS-CoV-2 resistance to remdesivir by in depth in vivo and in vitro studies.

Furthermore, the nucleoside analogue remdesivir can evade proofreading because its incorporation does not block elongation but only stalls RdRp after the addition of three more nucleotides [20]. Remdesivir was the first FDA-approved drug for the treatment of patients with COVID-19 [21], but its effectiveness is disputed [22]. Several other inhibitors targeting the nucleotide site of RdRp have been investigated in clinical trials, and they include Favipiravir, Ribavirin, and Galidesivir [23,24].

The aim of the present study was to structurally evaluate the impact of RdRp mu-tations found at baseline in patients treated with remdesivir that are associated with different degrees of antiviral response in vivo. The overall flowchart adopted in this work is schematically represented in the Supplementary Material, Figure S1.

## 2. Results

### 2.1. Patients’ Characteristics

In this study, we analysed the RdRp mutations from 39 patients with SARS-CoV-2 infection, all of whom were hospitalized for severe COVID-19 and treated for 5 days with the RdRp inhibitor remdesivir. Most patients (89.3%) had received a diagnosis of pneumonia at the time of hospitalization (Table S1). Overall, patients had a median (IQR) age of 66 (56.5–77) years and 56% were male. Notably, the vast majority of patients (89.3%) presented at least one comorbidity, with cardiovascular diseases representing the most frequently observed, followed by obesity, chronic respiratory diseases, and diabetes. At least one previous vaccination against SARS-CoV-2 was reported by 53.8% of the study population (Table S1).

The analysis of SARS-CoV-2 sequences revealed the presence of Delta variant in 64.1% of patients and Omicron in 28.2%, while the remaining 7.7% of patients harboured the B.1.177 viral strain.

In the overall population, Real-Time PCR assay for SARS-CoV-2 quantification in nasopharyngeal swab revealed median (IQR) CT values of 23.1 (20.7–25.7) for E target, 21.7 (19.2–24.0) for N target, and 24.9 (22.6–27.1) for RdRp target, thus indicating an intense viral replication before starting remdesivir therapy.

By analyzing SARS-CoV-2 viral load at T1 after completing 5 days of remdesivir therapy, the observed ∆CT values indicated an overall reduction in viral replication (median [IQR]∆CT of 5.6 [2.4–9.3], 5.4 [2.9–6.9], and 6.3 [2.8–9.1] for the E, N, and RdRp targets, respectively).

### 2.2. RdRp Mutational Profiles and Response to Remdesivir

In this study, we analysed the RdRp mutations from 39 hospitalized patients treated with the RdRp inhibitor remdesivir for SARS-CoV-2 infection. The mutational analysis of RdRp at treatment baseline revealed the circulation of six major mutational profiles. In particular, in 38.5% of patients, P323L was found to occur alone, while in the remaining patients, it was found in cluster with one or more additional mutations. Among them, the mutational pattern P323L+G671S was the most prevalent, characterizing 41% of patients, followed by the triple mutants P323L+G671S+L838I and P323L+G671S+M899I, which were detected in 10.3% and 5.1% of patients, respectively. Lastly, more complex mutational profiles characterized by the concomitant presence of 4 and 5 RdRp mutations, P323L+G671S+L838I+E58A and P323L+G671S+L838I+D738Y+K91E were revealed with a prevalence of 2.6% each.

By analyzing the variations in the cycle threshold values after the completion of a 5-day course of remdesivir treatment, with respect to the treatment baseline (∆CT), we observed a median (IQR) increase in the Ct for E, N, and RdRp targets of 5.5 (4.1–7.9), 6.3 (3.6–8.7), and 6.0 (5.4–8.6) in presence of the P323L alone, and of 8.0 (3.1–9.4), 5.4 (2.3–6.3), and 8.1 (3.9–8.8) with the couple P323L+G671S, indicating a relevant reduction in SARS-CoV-2 viral load in NS and, in turn, a good response to remdesivir treatment. Notably, the triple mutant P323L+G671S+M899I was associated with a decrease in the Ct for the three viral targets of −2.3, −0.3, and −1.9 at 7 days of remdesivir treatment, supporting an increase in SARS-CoV-2 viral load despite antiviral treatment and, thus, no response to remdesivir. Similarly, the patient carrying the complex mutational profile P323L+G671S+L838I+D738Y+K91E experienced a limited increase in the Ct for E, N, and RdRp targets of 1.8, 2.6, and 2.9 at 7 days of remdesivir treatment, suggesting a potential reduced response to remdesivir. For the other mutational profiles, we did not observe any relevant differences with respect to the mutant characterized by P323L alone. The overall data supports the need to explore the potential impact of RdRp mutations associated with a reduced response to remdesivir in order to understand their impact on both the structure of the viral protein and the binding of the drug.

### 2.3. Molecular Modeling

#### 2.3.1. Molecular Docking and Thermodynamics Analysis

**Figure 2:**
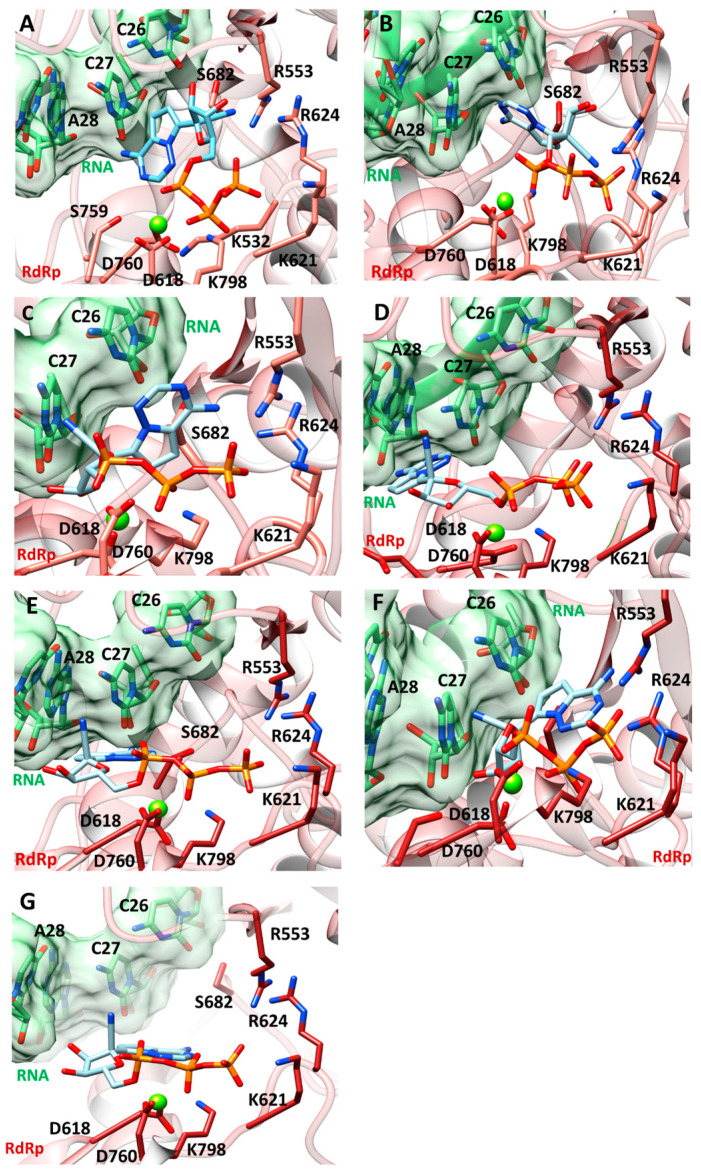
Best docking pose of remdesivir against: (**A**) WT (**B**) 1M (**C**) 2M (**D**) 3M_1_; (**E**) 3M_2_; (**F**) 4M; and (**G**) 5M. Remdesivir is depicted as light-cyan sticks, while RNA residues are shown as the light-green stick, surrounded by the light-green surface. The RdRp domain is shown as the dark-red ribbon, with the key interacting residues highlighted as dark-red sticks. Mg^2+^ ion is displayed as the green sphere. Explicit hydrogen atoms are omitted for reasons of clarity.

According to the clinically-evaluated SARS-CoV-2 RdRp mutations found in patients treated with remdesivir, and with the aim of evaluating the influence of the selected mutations on the binding affinity of remdesivir towards the polymerase domain, we analyzed the following complexes: the wild-type model (WT), P323L (1M), P323L+G671S (2M), P323L+G671S+L838I (3M_1_), P323L+G671S+M899I (3M_2_), P323L+G671S+L838I+E58A (4M), and P323L+G671S+L838I+D738Y+K91E (5M). We focused on simulating the moment immediately prior to the incorporation of the drug to the RNA, when it is still in the catalytic site and must be hydrolyzed in order to be incorporated as a nucleoside analogue within the RNA. The best obtained docking poses are shown in Figure 2.

As expected, remdesivir engaged crucial salt-bridge contacts between its triphosphate group and the positively-charged residues K621, K798, R553, and R555. However, a different positioning of the nucleoside moiety is observed in the mutants 2M (Figure 2C), 3M_1_ (Figure 2D), 3M_2_ (Figure 2E), 4M (Figure 2F), and 5M (Figure 2G) with respect to the WT (Figure 2A) and the most dominant single mutation P323L (1M) (Figure 2B). Here, in fact, the nucleoside moiety is oriented toward the RNA, but the nitrile group points toward the R555, R553, and R624, which are among the key binding residues [25]. On the contrary, in all of the mutated systems, the nucleoside part of remdesivir is oriented differently, with the nitrile group pointing far from the key binding residues. This is particularly evident in the 3M_2_ (Figure 2E) and the 5M (Figure 2G) systems.

With the aim of refining the molecular recognition studies and evaluating the free-energy contribution in the binding of remdesivir, we performed a thermodynamics analysis of the generated best pose for each RdRp-complex, thus obtaining the theoretical ΔG_bind_ values of the antiviral drug. By analyzing our thermodynamic results, we observed that some of the combined mutations enhance whilst some of the others reduce remdesivir affinity towards the polymerase domain. In particular, in 1M, 3M_2_, and 5M, we observed a decreased theoretical binding energy, thereby rationalizing the strong reduced response to remdesivir in patients bearing these mutational patterns, as confirmed by the increase in the SARS-CoV-2 viral load.

The docking scores and the theoretical ΔG_bind_ values for each model are reported in Table 1.

#### 2.3.2. Structural Effects on the Global Motions of the 3M_2_ and 5M Mutants

**Figure 3:**
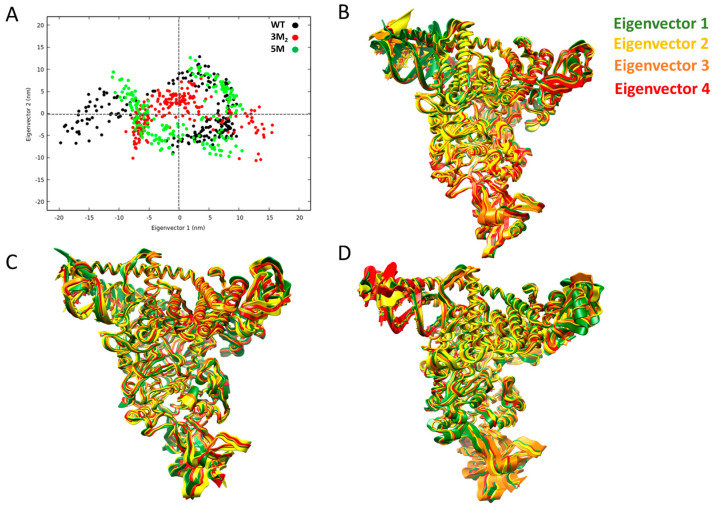
(**A**) Overlap between the 2d projection of the first two principal components (PCs) and the coordinates of the MDs trajectory: WT (black points), 3M_2_ (red points) and 5M (green points). (**B**–**D**) Slow relevant motions of the first four eigenvectors of the WT, 3M_2_ and 5M systems, respectively.

Unfortunately, docking calculations do not take fully into account the receptor flexibility. Thus, to give a complete explanation of the binding of remdesivir as well as the global allosteric effects of the mutants that are located far from the active site of RdRp on the structural changes of the RdRp, we have performed 200 ns of Molecular Dynamics simulations (MDs) followed by Principal Component Analysis (PCA) focusing on the structural analysis of the 3M_2_ and 5M mutations according to the evidence of the clinical studies regarding the loss of effectiveness of remdesivir in patients bearing such mutations. Furthermore, we have compared their global motions to those of WT in order to obtain a broader view and thus extract the dominant slow motions of the three systems. Firstly, the overlap of 2d projection of the first two highest Principal Components (PCs) was built on the WT, 3M_2_, and 5M systems. Generally, in such analysis, less phase space occupied is indicative of less motions and, therefore, of a more stable system. In our case, Figure 3A reveals that the 3M_2_ (red points) and the 5M (green points) systems occupy a less conformational phase space, thus representing less movements and, consequently, a higher stability. Conversely, the WT system (black points) occupies a more expanded conformational space with a more dispersive cluster as a result of a less stable complex and higher flexibility. Internal concerted motions of the three systems were further described looking at the first four highest eigenvectors (Figure 3B–D) obtained from the diagonalization of the covariance matrix on the overall trajectory strided every 1 ns. Particularly, we have observed the flexible motions that occur in the functional domains. It is well known, in fact, that Nsp7, Nsp8, and the holo-RdRp domain of Nsp12 facilitate the initiation and/or the elongation of viral RNA synthesis, and, in fact, the primer-extension processivity of SARS-CoV-2 RdRp is greatly increased in the presence of Nsp7 and Nsp8 [26]. The NiRAN domain possesses, instead, a self-nucleotidylating activity (NMPylation) [27]. We found that the flexible motions of these three Nsp7, Nsp8, and the RdRp functional domains are quite different in the three systems. As can be noted from Figure 3B, the largest motions (eigenvector 1) involve mainly the WT system and, specifically, the portion of the RNA template that is connected to the C-terminal region of the Nsp8T domain, while slighter motions occur in the RdRp domain involving the active site of remdesivir. On the contrary, in the 3M_2_/5M mutated systems, the C-terminal domain of Nsp8T is interested only by a few restricted movements. Thus, overall, the 3M2 mutations remarkably reduce the global motion patterns of the Nsp8 domain with respect to the WT where, instead, the binding of remdesivir induces a wider alteration. This finding is also in agreement with the previous observations of Byléhn et al., who proposed a third molecular mechanism of remdesivir that is characterized by RdRp complex destabilization due to the alteration of the Nsp8′s flexible regions [28]. In order to better verify which residues are mainly involved in the alteration, the RMSF profile on the same four PCs was built, highlighting a reduction of the fluctuations mainly in the RdRp domain of the 3M_2_ and 5M systems and also the Nsp8/RNA C-terminal regions (Supplementary Material, Figure S1).

#### 2.3.3. Representative Pharmacophore Models (RPM) of Remdesivir Extracted during MDs

As reported in other studies, the concept of pharmacophores [29] has become increasingly prevalent and strongly useful in computer-aided drug design techniques, highlighting the potential of this approach among research groups [30,31], even for the discovery of new molecules or for the repositioning of already existing drugs against SARS-CoV-2 [32]. A novel 3D pharmacophore analysis method, implemented in the LigandScout Expert version [33], allows us to gather all of the “ligand-receptor” interactions occurring throughout a complete MD trajectory. Here, the structure-based pharmacophore, which is specialized to detect ligand-protein interactions, was applied to the MD trajectories of the two mutated 3M_2_ and 5M systems, and then compared with the WT model.

**Figure 4:**
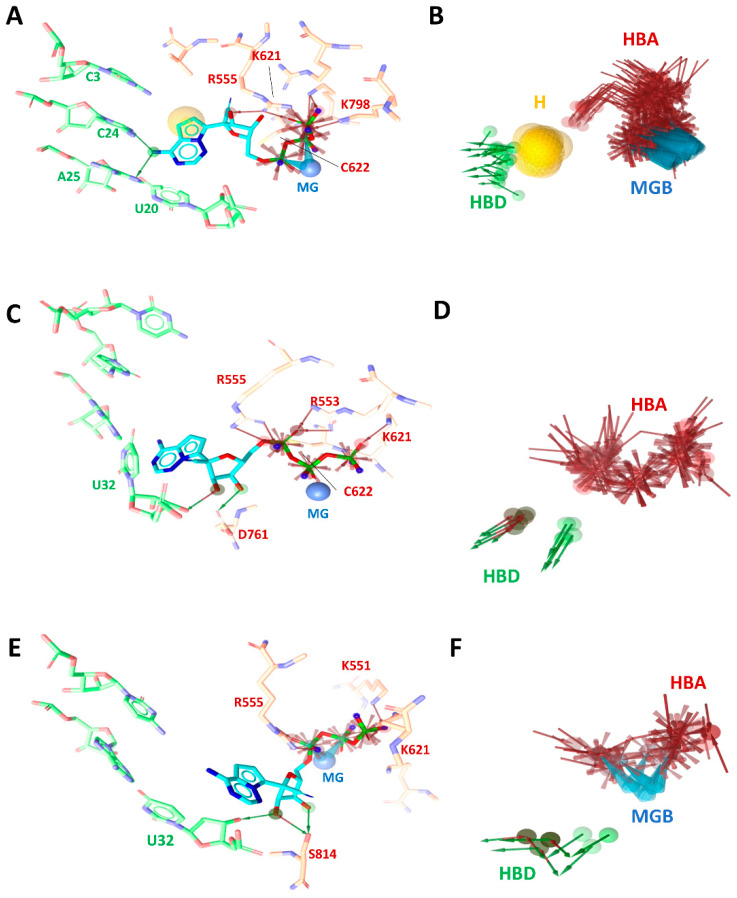
3D pharmacophore models and representative pharmacophore models (RPMs) of remdesivir during 200 ns of MDs in (**A**,**B**) WT, (**C**,**D**) 3M_2_, and (**E**,**F**) 5M complexes. The main residues involved in the binding with remdesivir are labeled and highlighted in stick.

For the WT model and the two mutated systems, 3M_2_ and 5M, the representative pharmacophore models (RPMs) were selected after clusterization (Figure 4B,D,F). Results showed a different interaction pattern of the remdesivir in the three models (Figure 4A,C,E). Statistical analysis of the RPMs are instead reported in Tables S2–S4 of the Supplementary Material. RPMs were extracted in order to understand both the dynamic behavior and the chemical features of remdesivir in all of the systems (200 frames for each simulation). Interestingly, from Tables S2–S4, the high difference in the interaction occurrence (percentage) of the remdesivir in the WT model can be observed, especially if compared with those of 3M_2_ and 5M complexes, in which we observe few interactions between the ligand, the RNA, and the main residues of both the 3M_2_ and the 5M models. In particular, in the WT model, the differences showed a high appearance of both the hydrogen bond donor (HBD) and acceptor (HBA) with C24 and C25 RNA-bases, as well as R555, K621, K798, and C622 RdRp residues. Furthermore, a strong interaction was highlighted between the C23 base and remdesivr, which was absent in the other systems. Magnesium ions are essential in the catalytic site because they may serve as catalytic ions near the active site [34]. In the WT model, the interaction between remdesivir and the magnesium ion was more present than in the 3M_2_ and 5M models, where we observed a lower occurrence percentage.

## 3. Discussion

In this study, the impact of RdRp genetic profiles on modulating virological response to Remedesivir was evaulated in a set of 39 hospitalized patients with a RdRp sequence available at the time of starting the antiviral treatment. As expected, all of the RdRp sequences were characteized by the presence of the P323L mutation that is located at the NiRAN/RdRp interface, which, nowadays, has almost replaced the WT sequence [35]. We have previously demonstrated, by molecular dynamics simulations, that P323L increased the number of HBs between RdRp and NSP-8, a cofactor that is part of the replication complex along with RdRp, suggesting a stabilized interaction between these two proteins and, in turn, the fixation of this mutation in a viral population [36].

The P323L was accompanied by the presence of simultaneous mutation patterns, here initialed as 2M, 3M_1_, 3M_2_, 4M, and 5M. All of the patients were treated with the RdRp inhibitor remdesivir, and, interestingly, it turned out that the patients bearing the concomitant mutations P323L-G671S-M899I (3M_2_) and P323L-G671S-L838I-D738Y-K91E (5M) in the RdRp machinery of SARS-CoV-2 showed a delayed or up to no response to remdesivir, which suggests that the progressive enrichment of mutations in RdRp can play a role in jeopardizing remdesivir efficacy, thereby promoting the onset of drug resistance. In keeping with our findings, another previous study supported the association of mutational patterns, including L838I, that are present at the remdesivir baseline, with a reduced or no response to the drug in vivo [37]. On the contrary, no relevant differences with respect to the P323L alone or the WT were observed in the remaining concomitant mutations pattern. Given such clinical evidence, the reduced efficacy of remdesivir associated with the Nsp12 mutations was evaluated using a combination of computational approaches that considered remdesivir in its preincorporation (triphosphate) state, and the aim of this was to elucidate the effect of the mutations on the dynamic behaviors of the functional domains and understand the molecular basis underlaying such drug resistance. Specifically, docking and thermodynamic evaluations revealed very different binding modes of remdesivir among the variants, with a significant decrease in the ΔG_bind_ value in the 1M, 3M_2_, and 5M variants with respect to the WT. This is coherent with a different orientation, especially of the nucloside moiety and the nitrile group, which, in some cases, is further apart from the key interacting residues of the RdRp domain. In order to investigate these effects in more depth, since such RdRp mutations are distant from the RNA/remdesivir active site, we elucidated the allosteric effects on the global motions through Principal components analysis (PCA). This analysis revealed that the mutation pattern of 3M_2_ and 5M systems does not signficantly alter the correlated motions of RdRp, which is in contrast to the WT system, in which a significant alteration in the magnitude of the movements was observed in the first five normal modes, particularly at the interface of the Nsp8 and the RNA, which is important for the polymerase activity. This hypothesis is further confirmed by the dynamic pharmacophore approach, in which a reduction of the interaction patterns of remdesivir with RNA was found in the 3M_2_ and the 5M variants.

Despite our findings suggesting a potential role of some specific RdRp mutational patterns in altering the binding of remdesivir to its viral target and, in turn, modulating its efficacy against SARS-CoV-2, the clinical impact of the computational predictions of this study need to be validated in a larger study population. Moreover, further in vitro and in vivo experiments are also necessary in order to corroborate the in silico analyses and, finally, to elucidate the relevance of the detected RdRp mutations for the effectiveness of antiviral therapy based on RdRp inhibitors.

Overall, despite these limitations, this study offers valuable information on the potential evolution of mutations that could lead to a reduced efficacy of remdesivir, highlighting viral factors and molecular mechanisms that could contribute to this reduced efficacy, and thereby providing a foundation for monitoring early signs of potential resistance to this drug. Overall, this can also represent a proof of concept study that is exportable to the setting of other anti-SARS-CoV-2 drugs. Indeed, our approach combining clinical and structural data will be critical for enlarging the current knowledge on both SARS-CoV-2 mechanisms of resistance (both natural and drug-induced) and the critical issues for setting up more personalized and optimized treatment strategies against COVID-19 with higher chances of therapeutic success, and, in turn, with a reduced risk of evolution towards severe clinical events.

## 4. Materials and Methods

### 4.1. Study Population and Mutational Analysis of RdRp

In this study, we included all SARS-CoV-2 positive individuals consecutively hospitalized for severe COVID-19 at the University Hospital of Rome Tor Vergata in Central Italy from September 2020 to March 2022 (N = 39). All of the patients met AIFA criteria for eligibility to an early treatment and, on this basis, were treated with remdesivir for a duration of 5 days, initiated just after receiving the diagnosis of SARS-CoV-2 infection.

Nasopharyngeal-swabs (NS) at the time of starting remdesivir were used to obtain the full-genome sequence of SARS CoV-2 and to analyse SARS-CoV-2 RdRp mutational profiles by using the COVIDSeq Assay (96 samples) index 1 (Illumina inc., San Diego, CA 92122, USA.) according to the manufacturer’s instructions.

A refined bioinformatics approach was then applied to assign SARS-CoV-2 clade and lineage and to define RdRp mutational profiles. In particular, the RdRp mutations were defined according to the sequence of RdRp protein using the NC_045512.2 SARS-Cov-2-Wuhan-Hu-1 isolate as the reference sequence. The intrapatient prevalence of each RdRp mutation was calculated in the overall population, and all mutations with an intrapatient prevalence >20% (major mutations) were considered. In detail, the FASTQ obtained for each sample after sequencing was analyzed using the Genome Detective Virus Tool [38] in order to assign a taxonomic name and mutations. In addition, quality control of the raw data were performed by Trimmomatic [39] software in order to remove adapters, PCR primers, and poor quality reads. Fastq files were then analyzed with VirVarSeq software [40] using SARS-CoV-2 consensus (NC_045512.2) as reference. Only variants with frequency >20% were retained for further analysis. For each sample, consensus sequences were generated by using quasitools [41], and were uploaded on nextstrain in order to assign the clade [42] and on Pangolin lineages to assign the variant [43].

The study protocol on the collection of samples and the sequencing of SARS-CoV-2 was approved by the Ethics Committee of Fondazione PTV Policlinico Tor Vergata (register number46/20, 26 March 2020) and conducted in accordance with the 1964 Declaration of Helsinki. The individuals allowed the viral sequencing for surveillance, and the samples were previously anonymized, according to the requirements of the Italian Data Protection Code (leg. decree 196/2003). Demographic, epidemiological, and clinical information were obtained retrospectively, and were collected in a pseudonymized database.

### 4.2. Evaluation of the Impact of RdRp Mutations on the Response to Remdesivir

At both baseline (T0) and at day 6 after completing remdesivir treatment (T1), Real-Time-PCR results based on 3 viral gene targets (envelope (E), nucleocapsid (N), and RNA-dependent-RNA-polymerase (RdRp)) were also obtained by AllplexTM SARS-CoV-2 Assay (Seegene). The variation in the cycle threshold (∆CT) values in NS between T1 and T0 were used as a measure of the variation of SARS-CoV-2 load after treatment, and were then used to evaluate the response to remdesivir. Lastly, the variation in SARS-CoV-2 load was analysed in patients according to the presence of the different RdRp mutational profiles.

### 4.3. SARS-CoV-2 Polymerase Model Preparation

Due to the completeness of the entire replication machine both in terms of amino acids and of the structural chains making up the entire viral RNA replication complex, which requires the presence of Nsp12, Nsp7, and Nsp8, the cryo-EM model with the PDB ID 7ED5 was selected as a reference structure (Figure 1). Here, the triphosphate form of the original AT-527 ligand in the catalytic site of RdRp is still not incorporated in the RNA strand, and is ready for the hydrolysis reaction of pyrophosphate groups (AT9) [44].

The original ligand was extracted and the apo-form of the target was optimized by using the Protein Preparation Wizard tool of Maestro suite (Schrödinger Release 21.4) [45] and OPLS_2005 as force field [46] in order to delete complexed water molecules, adding hydrogen atoms, correcting the connectivity, and generating the exact protonation states at pH 7.4. Missing side chains and loops were built using Prime [47].

### 4.4. Preparation of the SARS-CoV-2 Polymerase Mutant Models

Starting from the WT model, all of the mutants were built using the Maestro Graphic Interface (Schrödinger-Version 21.4) [48]. In particular, according to the mutations found in patients in the clinical assays, six models were built: P323L (1M), P323L-G671S (2M), P323L-G671S-L838I (3M_1_), P323L-G671S-M899I (3M_2_), P323L-G671S-L838I-E58A (4M), and P323L-G671S-L838I-D738Y-K91E (5M).

### 4.5. Molecular Docking and Thermodynamics Analysis

To assess the accuracy and reliability of our docking protocol, we performed re-docking calculations by using the Standard Precision (SP) algorithm of Glide software v.6.7, generating 10 poses per ligand [49]. The Root Mean Square Deviation (RMSD) between the cryo-EM pose of the ligand and that of the best-scored docking pose was calculated, resulting in a good overlap (RMSD value equal to 1.94 Å). After refinement, the receptors were submitted to 10,000 steps of MacroModel energy minimization [50], which was carried out using the OPLS_2005 force field [46]. All of the optimized structures were used as reference models to perform docking studies of remdesivir by adopting the abovementioned protocol and generating 25 poses per ligand. The grid generation step was performed by centering the grid box on the active site of RdRp by considering the triphosphate form of AT-527 as a pointer. The best-scored docking poses were further submitted to thermodynamic analysis using the Multi-Ligand Bimolecular Association with the Energetics (Embrace) tool, with VSGB as the solvation model and OPLS_2005 as the force field [46,50].

### 4.6. Molecular Dynamics Simulations (MDs)

MDs of the Nsp12/Nsp8/Nsp7/RdRp/NiRAN and the RNA primer-template complexed to remdesivir were run using the GROMACS ver.2020.6 simulation package [51]. Specifically, according to the most impactful clinical evidences on patients under treatment with remdesivir, and in order to clarify the molecular mechanism underlying the loss of efficacy of remdesivir, three systems were simulated: WT, 3M_2_, and 5M. Remdesivir coordinates were taken from the best-scored docking pose of each system. The partial atomic charges of remdesivir were determined by the restrained electrostatic potential (RESP) fitting procedure [52] that was implemented in the Antechamber program of AmberTools22 software package [53]. Prior to the RESP fitting, the ligand electrostatic potential (ESPs) was computed at the HF/6-31G* level with the Gaussian16 package [54]. Finally, the General Amber Force Field (GAFF2) [55] was used to assign atom types and bonded parameters to the ligand, and then the WT, 3M_2_, and 5M systems were converted into appropriate GROMACS topology file format using the acpype script [56]. The ff99SB amber force field was used in all simulations to parametrize the systems [57]. The TIP3P water model in a truncated octahedron box was used to solvate the systems, and counterions were added to neutralize the net charge. The MDs protocol included a first minimization run of 1 ns with the conjugate gradient algorithm in order to minimize water molecules and hydrogen atoms by applying a force constant of 1000 kj/mol on both protein, RNA and ligand heavy atoms. A second minimization step of 1 ns was run without any constraint, and then 5 ns of equilibration step in the canonical NVT ensemble with a Berendsen-thermostat with a temperature coupling constant of 1.0 ps was run by increasing the temperature from 0 to 300 K. A second 5 ns of equilibration step in the NPT ensemble was instead run with the Berendsen barostat at 1 atm. Finally, 200 ns of MDs production runs were carried out using a constant pressure ensemble (NPT). All simulations were performed using periodic boundary conditions and a 2 fs time step. Long-range electrostatic interactions were calculated using the Particle Mesh Ewald (PME) method with a non-bonded cut-off of 10 Å and the LINCS algorithm [58] to constrain all bond lengths. MDs results analysis was carried out using the gmx rms, gmx rmsf commands of GROMACS.

### 4.7. Principal Component Analysis (PCA)

Functionally relevant slow motions were analyzed by means of PCA. Specifically, diagonalization of the covariance matrix was performed using the gmx covar tool of GROMACS on the backbone atoms of both the Nsp12/Nsp8/Nsp7/RdRp/NiRAN and the RNA of the WT, 3M_2_, and 5M systems to compare the effect of the mutants with respect to the WT. Then, the motion of the protein was identified by projecting the first four eigenvectors with the gmx anaeig module. Graphs were plotted using the Gnuplot tool.

### 4.8. Pharmacophore Model Generation along the MDs

The MDs trajectories, strided every 1 ns, of the three mutated systems (WT, 3M_2_, and 5M), complexed to remdesivir, were used to create representative pharmacophore models (RPMs). RPMs were extracted to understand the dynamic behavior and the chemical features of remdesivir in all of the systems (200 frames for each simulation). This is an innovative technique to reveal the interaction pattern of a ligand in the binding pocket. The advanced version of LigandScout 4.4.9 software [33] was used for the extended pharmacophore investigation on the trajectories of the MDs.

## Figures and Tables

**Table 1:** The best Glide score (G-score) and Embrace ΔG_bind_ values obtained after docking simulations of remdesivir against WT and all of the analysed mutated complexes of RdRp. The G-scores and Embrace energy values are expressed in kcal/mol.

Rdrp Model	G-Score	ΔG_bind_
WT	−8.63	−122.70
1M	−8.74	−34.50
2M	−8.58	−119.05
3M_1_	−8.72	−129.41
3M_2_	−8.81	−84.78
4M	−8.44	−125.56
5M	−8.76	−96.74

## Data Availability

Data is contained in the article and Supplementary Material.

## Supplementary Materials

The following supporting information can be downloaded at: https://www.mdpi.com/article/10.3390/ph16081143/s1: Figure S1: flowchart schematically representing the overall approach adopted in the study. Figure S2: RMSF plot built on the four PCs revealing the changes in the fluctuations of 3M_2_ and 5M mutated residues with respect to the WT. Table S1: Demographic characteristics of the study population. Tables S2–S4: Statistical analyses of remdesivir pharmacophore interaction patterns during 200 ns of MDs in the Wild-type (WT), 3M_2_, and 5M systems, respectively.
