# Supplementary material for: Molecular and Structural Aspects of Clinically Relevant Mutations of SARS-CoV-2 RNA-Dependent RNA Polymerase in Remdesivir-Treated Patients
Source: Pharmaceuticals (Basel). 2023 Aug 12;16(8):1143. doi: 10.3390/ph16081143 (PMC10459223; doi:10.3390/ph16081143)
Supplement: Supplementary file 1 [file pharmaceuticals-16-01143-s001.zip › pharmaceuticals-2530899-supplementary.pdf]

SUPPLEMENTARY MATERIAL

FOR

Molecular and structural aspects of clinically relevant mutations of SARS-CoV-2 RNA-dependent RNA polymerase in Remdesivir treated patients

Carmen Gratteri[1], Francesca Alessandra Ambrosio[2], Antonio Lupia[3,4], Federica Moraca[4,5,*], Bruno Catalanotti[5], Giosuè Costa[1,4], Maria Bellocchi[6], Luca Carioti[6], Romina Salpini[6], Francesca Ceccherini-Silberstein[6], Simone La Frazia[7], Vincenzo Malagnino[8], Loredana Sarmati[8], Valentina Svicher[7], Sharon Bryant[9], Anna Artese[1,4,*] and Stefano Alcaro[1,4]

[1] Dipartimento di Scienze della Salute, Università degli Studi "Magna Græcia" di Catanzaro, Campus "S. Venuta", Viale Europa, 88100, Catanzaro, Italy; carmen.gratteri@studenti.unicz.it (C.G.), gcosta@unicz.it (G.C.), artese@unicz.it (A.A.), alcaro@unicz.it (S.A.)

[2] Dipartimento di Medicina Sperimentale e Clinica, Università degli Studi "Magna Græcia" di Catanzaro, Campus "S. Venuta", Viale Europa, 88100 Catanzaro, Italy; ambrosio@unicz.it (F.A.A.).

[3] Dipartimento di Scienze della vita e dell'ambiente, Università degli Studi di Cagliari, Cittadella Universitaria di Monserrato, 09124 Cagliari, Italy; antonio.lupia@unica.it (A.L.).

[4] Net4science srl, Università degli Studi "Magna Græcia" di Catanzaro, 88100, Catanzaro, Italy; antonio.lupia@unica.it (A.L.); federica.moraca@unina.it (F.M.); gcosta@unicz.it (G.C.), artese@unicz.it (A.A.), alcaro@unicz.it (S.A.).

[5] Dipartimento di Farmacia, Università degli Studi di Napoli "Federico II", Via D. Montesano 49, 80131, Napoli, Italy; federica.moraca@unina.it (F.M.); bruno.catalanotti@unina.it (B.C.).

[6] Dipartimento di Medicina Sperimentale, Università Tor Vergata di Roma, Via Montpellier, 1, 00133 Roma, Italy; maria.bellocchi@gmail.com (M.B.); luca.carioti@yahoo.com (L.C.); rsalpini@gmail.com (R.S.); ceccherini@med.uniroma2.it (F.C.S.).

[7] Dipartimento di Biologia, Università Tor Vergata di Roma, Via della Ricerca Scientifica, 1, 00133 Roma, Italy; simone.la.frazia@uniroma2.it (S.L.F.); valentina.svicher@uniroma2.it (V.S.).

[8] Dipartimento di Medicina dei Sistemi, Università Tor Vergata di Roma, Via Montpellier, 1, 00133 Roma, Italy; vincenzo.malagnino@uniroma2.it (V.M.); loredana.sarmati@uniroma2.it (L.S.).

[9] Inte:Ligand GmbH, Mariahilferstrasse 74B/11, 1070, Vienna, Austria; bryant@inteligand.com (S.B.).

* Correspondence: artese@unicz.it; Tel.: +39 0961 3694297; federica.moraca@unina.it; Tel.: +39 081 678551

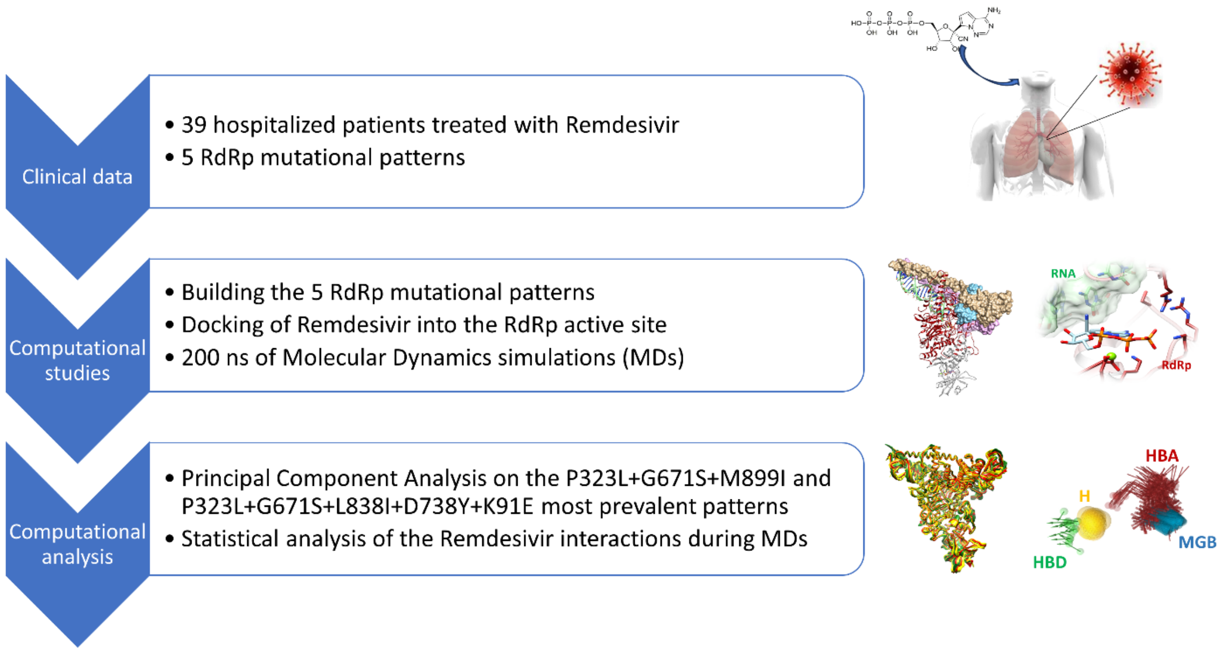

Figure S1. flowchart schematically representing the overall approach adopted in the study.

Table S1. demographic characteristics of the study population.

**Patients' Characteristics (N=39)**	
Male, N (%)	22 (56.4)
Median (IQR) Age, years	66 (56.5-77)
Pneumonia diagnosis, N (%)	35 (89.7)
Comorbidities/Risk factors, N (%)	
Presence of at least 1 comorbidity	35 (89.7)
Median (IQR) number of comorbidities per patient	3 (2-5)
Obesity (BMI>30)	12 (30.8)
Cardiovascular Diseases	25 (64.1)
Chronic respiratory diseases	10 (25.6)
Diabetes	9 (23.1)
Cancer	7 (17.9)
Hepatic diseases	2 (5.1)
Vaccination, N (%)	21 (53.8)
SARS-CoV-2 variant, N (%)	
Delta	25 (64.1)
Omicron Ba.1	9 (23.1)

B.1.177	3 (7.7)
Omicron BA.2	2 (5.1)
Baseline Ct at starting Remdesivir therapy (T0), Median (IQR)	
E target	23.1 (20.7-25.7)
N target	21.7 (19.2-24.0)
RdRp target	24.9 (22.6-27.1)
Delta Ct after completion of 5-day Remdesivir therapy , Median (IQR)	
E target	5.6 (2.4-9.3)
N target	5.4 (2.9-6.9)
RdRp target	6.3 (2.8-9.1)

Abbreviations: IQR: interquartile range, BMI: Body mass index , RdRp: RNA-dependent RNA polymerase

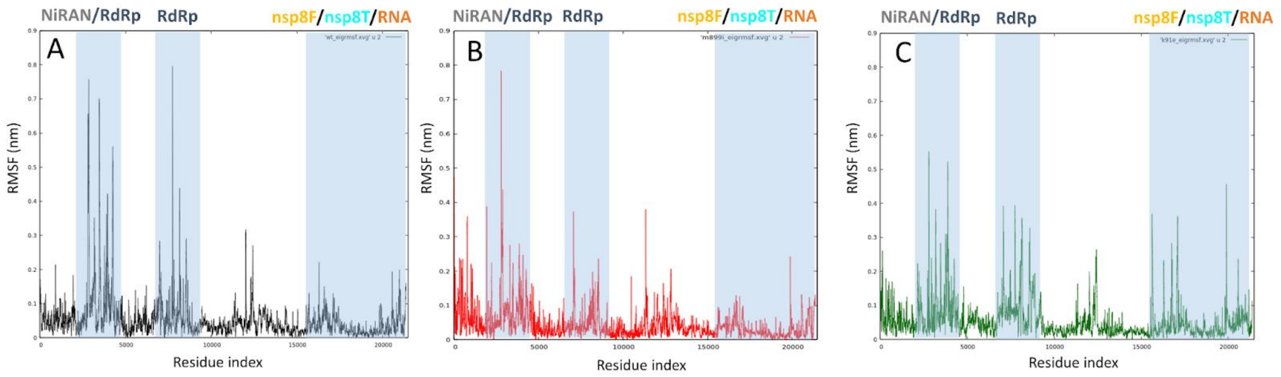

Figure S2. RMSF plot built on the four PCs revealing the changes in the fluctuations in **(A)** WT, **(B)** 3M2 and **(C)** 5M mutated residues with respect to the WT.

Table S2. Statistical analysis of Remdesivir interaction patterns during 200 ns of MDs in the Wild-type (WT) system. AR (Aromatinc Ring interaction), HBA (Hydrogen Bond Acceptor), HBD (Hydrogen Bond Donor), NI (Negative Ionizable), MGB (Coordination bond with Magnesium ion). RNA and RdRp interacting residues are colored in green and dark-red, respectively.

WT	A688	R553	R555	R624	C622	K545	K551	K621	K798	MG933	RA25	RC23	RC24	RU3-20	S682	T687	V557	Appearance (%)
AR						0%												0%
AR						0%												0%
H	1%											56%	19%			19%	0%	69%
HBA					6%													7%
HBA		0%	85%															85%
HBA		12%	0%															12%
HBA					1%													2%
HBA			2%				0%		22%									26%
HBA									37%									37%
HBA		0%			93%			44%	0%									96%
HBA		19%	1%				25%	52%	2%									69%
HBA		48%	81%				1%	3%	1%									95%
HBA		0%	94%				28%	0%	3%									97%
HBA				16%											2%			14%
HBA																0%		0%
HBD					0%													1%
HBD											21%		73%	0%	0%			73%
MGB										100%								100%
MGB										100%								100%
NI		2%	1%				12%	73%	56%	100%								100%
NI			1%						1%	100%								100%
NI		99%	93%				57%	75%	54%	34%								100%

Table S3. Statistical analysis of Remdesivir interaction patterns during 200 ns of MDs in the P323L+G671S+M899I (3M2) system. AR (Aromatinc Ring interaction), HBA (Hydrogen Bond Acceptor), HBD (Hydrogen Bond Donor), NI (Negative Ionizable), MGB (Coordination bond with Magnesium ion). RNA and RdRp interacting residues are colored in green and dark-red, respectively.

$3M_2$	A688	R553	R555	N691	D760	D761	C622	K551	K621	K798	MG933	RA28	RC27	RU3-32	S682	S759	Appearance (%)
AR			0%														0%
H	0%												0%				3%
HBA			36%														34%
HBA														52%		0%	53%
HBA														19%			19%
HBA			16%				32%										43%
HBA							2%	4%									7%
HBA		69%	31%					9%									87%
HBA		30%	89%														76%
HBA		4%					1%	3%		2%							12%
HBA			0%					8%		5%							14%
HBA							3%										3%
HBA		8%					9%	14%	2%	15%							44%
HBA		7%					3%	21%	8%	20%							55%
HBA		12%					0%	29%	3%	14%							55%
HBA			0%											6%		1%	9%
HBA			1%											0%			2%
HBA				25%												3%	28%
HBD						1%								44%		5%	53%
HBD					1%	48%								14%		6%	65%
HBD							2%					1%	2%		0%	0%	15%
MGB											64%						64%
NI		62%	92%					20%			57%						100%
NI		47%	0%					32%		36%	100%						100%
NI		54%						86%	16%	62%	99%						100%

Table S4. Statistical analysis of Remdesivir interaction patterns during 200 ns of MDs in the P323L+G671S+L838I+D738Y+K91E (5M) system. AR (Aromatinc Ring interaction), HBA (Hydrogen Bond Acceptor), HBD (Hydrogen Bond Donor), NI (Negative Ionizable), MGB (Coordination bond with Magnesium ion). RNA and RdRp interacting residues are colored in green and dark-red, respectively.

5M	R553	R555	D618	D761	C813	L758	K545	K551	K621	K798	MG933	RA28	RC27	RU3-32	S814	Appearance (%)
AR		16%														16%
AR							0%									0%
H						0%										1%
HBA		97%						0%								67%
HBA					0%									28%	97%	99%
HBA															18%	19%
HBA		28%							0%							23%
HBA		23%														24%
HBA		77%						1%								73%
HBA								0%		30%						31%
HBA										1%						1%
HBA									81%	5%						84%
HBA	0%	4%						11%	24%	20%						55%
HBA		12%						21%	17%	22%						69%
HBA		15%						11%	15%	33%						69%
HBA														5%	6%	11%
HBA															1%	2%
HBD				0%										90%	74%	95%
HBD			39%	4%											76%	97%
HBD												28%	0%			28%
MGB											98%					98%
MGB											100%					100%
NI	0%	100%						3%	22%		100%					100%
NI		68%						5%	90%	96%	100%					100%
NI	0%	60%						84%	91%	97%	3%					100%
